# Catalytic Synthesis of α-Oxoketene *S,S*-Acetals in a Wet Ionic Liquid [Bmim]Cl/H_2_O Homogeneous System

**DOI:** 10.3390/molecules16064500

**Published:** 2011-05-27

**Authors:** Ping Yu, Yuangang Zu, Yujie Fu, Thomas Efferth

**Affiliations:** 1 Key Laboratory of Forest Plant Ecology, Ministry of Education, Northeast Forestry University, Harbin 150040, China; 2 Engineering Research Center of Forest Bio-preparation, Ministry of Education, Northeast Forestry University, Harbin 150040, China; 3 Department of Pharmaceutical Biology, Institute of Pharmacy and Biochemistry, University of Mainz, Mainz 55128, Germany

**Keywords:** 1,3-dicarbonyl compounds, α-oxoketene *S,S*-acetals, [Bmim]Cl, water

## Abstract

A clean, practical and environmentally friendly synthesis in a homogeneous system has been developed for α-oxoketene *S,S*-acetals. A mixture of [Bmim]Cl and water was used as medium. The best economical and practical molar ratio of [Bmim]Cl to substrate was 4 to 1. With various types of 1,3-dicarbonyl compounds as substrate, the corresponding α-oxoketene *S,S*-acetals have been prepared under mild reaction conditions with yields of 53–74% after purification with silica gel column. [Bmim]Cl/water can be recycled several times.

## 1. Introduction

α-Oxoketene *S,S*-acetals are known as versatile intermediates for the preparation of various functional groups such as acetylenes [1], substituted furans [2], thiophene derivatives [3] and highly substituted phenols [4,5]. The synthesis of an α-oxoketene *S,S*-acetal albeit in low yield was first reported by Kelber and co-workers in 1910 using potassium hydroxide as base [6]. In 1988, Choi *et al.* [7] successfully prepared α-oxoketene *S,S*-acetals directly from ketones in good yields by choosing potassium carbonate as weak base in *N,N*-dimethylformamide. However, environmental pollution and post-processing difficulties of *N,N*-dimethylformamide led to some drawbacks. In 2005, Ouyang *et al*. [8] successfully prepared α-oxoketene *S,S*-acetals in good yields using water as medium, and TBAB as phase transfer catalyst in a solid-liquid system. However, uneven mixing is a frequent phenomenon in solid-liquid systems, especially in the amplification reaction. In order to overcome this issue, we have chosen ionic liquids as phase transfer catalyst [9] and cosolvent, water as medium to synthesize α-oxoketene *S,S*-acetals. The most apparent advantage was that ionic liquids/H_2_O homogeneous system could efficiently avoid uneven mixing of starting material mixtures. To the best of our knowledge, the present investigation is the first report of such a synthesis method.

Ionic liquids had been generally studied as excellent solvents due to their unique physical and chemical properties [10]. As an imidazolium ionic liquid, [Bmim]Cl has been widely used in many reactions such as elimination reactions [11], one-pot synthesis of α-aminophosphonate derivatives [12], oxidations [13,14], Friedel-Crafts acylations [15], methanolysis of polycarbonates [16], *S*-methylation reactions [17] and Ferrier rearrangements [18]. Its clean, green, high yield, no vapor pressure and recyclability characteristics were evidenced in these reactions. More interesting is the fact that [Bmim]Cl in some reactions has the feature of decreasing the reaction temperature to room temperature from 100–300 °C [19], promoting the reactivity of metal catalysts [20], greatly reducing the reaction times [21], high selectivity [15,17,18] and less toxicity [17].

Water and ionic liquids mixtures were termed “wet ILs” [22] and are used as single-phase or multi-phase systems. A wide range of applications have been realized in recent years such as Knoevenagel condensation [23], Diels–Alder cycloadditions [24], synthesis of organophosphorus compounds [25] and Biginelli-type reactions [26]. Some studies have indicated that the presence of water has a great impact on ionic liquids [27,28]. These previous achievements encouraged us to establish an environmentally friendly synthesis method for α-oxoketene *S,S-*acetals in an ionic liquid/water system. The choice of ionic liquid, the ratio of ionic liquid to starting material, recycle times and other factors were investigated and optimized to achieve a green, moderate yield synthesis of α-oxoketene *S,S*-acetals.

## 2. Results and Discussion

### 2.1. Choice of Ionic Liquids

A weak alkaline system is needed for synthesis of α-oxoketene *S,S*-acetals rather than strong alkali to prevent side reactions. Considering the extensive applications and especially the many favorable features and its lower price [Bmim]Cl has been chosen for this investigation. Another frequently used and cheap pyridine ionic liquid [Epy]BF_4_ has also been chosen for the investigation. The alkalinity was regulated by addition of the weak base potassium carbonate. Water, the ionic liquids [Epy]BF_4_, [Bmim]Cl, [Epy]BF_4_/water and different [Bmim]Cl/water ratios were used as solvents, respectively. Results are shown in Table 1.

No reaction product was detected when using water alone (Entry 1). When using [EPy]BF_4_, [Bmim]Cl respectively, all materials appeared as a solid phase without further reactivity (Entry 2, 3), indicating that the reaction did not take place in ionic liquids. However, under the systems of ionic liquids [EPy]BF_4_, [Bmim]Cl and water (wet ILs), the reactions took place with good yields. Acetoacetylaniline 5 mmol (taken as 1 equiv. relatively), potassium carbonate (2.5 equivs.), carbon disulfide (1.2 equivs.) and 1,2-dibromoethane (1 equiv.) in [EPy]BF_4_/water (10 equivs./15 mL) led to good reactivity and 71% yield (Entry 4). However, the reaction time took up to 15 hours. Then, the reaction of acetylacetanilide (1 equiv.) in [Bmim]Cl/water (10 equivs./15 mL) was attempted in accordance with the experimental procedures. Interestingly, a homogeneous system was obtained giving 73% yield of product after 5 hours (Entry 5). It was reported that [Bmim]Cl plays an important role in accelerating the reaction rate [18,21]. We assume that [Bmim]Cl also played the same role in our reactions.

### 2.2. The Ratio of [Bmim]Cl to Substrate

To optimize the reaction conditions, several reactions were carried out changing the molar ratio [Bmim]Cl to **1a** from 50/5 to 0.1/5. As shown in Table 1 (Entry 5), the effect of using 10 equivs. [Bmim]Cl was excellent. The yields and reaction time were monitored while the amount of [Bmim]Cl was reduced successively to 0.02 equivs. A reduced molar quantity of [Bmim]Cl of 4 equivs. (Entry 8) still resulted in successful dissolution of the starting material. The reaction effect was almost the same as Entry 5. As the molar quantity of [Bmim]Cl was reduced to 2 equivs. (Entry 9), a small amount of insoluble starting material was observed. Nevertheless, no obvious yield decline was measured. With continuous reduction of the amount of [Bmim]Cl, the reaction time was prolonged, accompanied by more amount of unreacted starting material. The explanation is that the amount of [Bmim]Cl as catalyst in the system was relatively low, which consequently led to low reaction rates and conversions. In addition, without enough [Bmim]Cl to dissolve the raw material in water, uneven stirring in solid-liquid system resulted in low conversion. Therefore, the best molar ratio of [Bmim]Cl to substrate was 4 to 1 (Entry 8).

### 2.3. Recycling of the [Bmim]Cl/water

The feasibility of recycling ionic liquids/H_2_O represents an important topic and performance criterion of any industrial process. The proportion of [Bmim]Cl and water did not change much after filtering off the products. This prompted us to investigate the recycling of the wet ionic liquid. After isolated the product by filtration, the residual [Bmim]Cl/H_2_O of Table 1, Entry 8 was collected without any treatment for the reuse. The reaction of acetoacetylaniline (1 equiv.), carbon disulfide (1.2 equivs.) and 1,2-dibromoethane (1 equiv.) while reusing the wet ionic liquid was analyzed. The corresponding α-oxoketene *S,S*-acetal was produced in a yield of 70% (Table 2, Entry 1). After filtering the product by the same process as described above, the residual [Bmim]Cl/H_2_O was reused for a third, fourth and fifth time. The results of subsequent studies (Table 2) indicated that the yield did not decline even in the fifth run with reused [Bmim]Cl/water. This finding implied that wet ionic liquid can be recycled, and the catalytic activity of [Bmim]Cl is not decreased.

### 2.4. The Synthesis of Different α-oxoketene S,S-acetals

To extend the applicability of this protocol, a series of substrates **1a**-**1e** were subjected to the reaction using the optimized conditions (Table 1, Entry 8). The results are summarized in Table 3.

The yields of cyclic α-oxoketene *S,S*-acetals (e.g., Entry 3) were higher than for acyclic α-oxoketene *S,S*-acetals (Entries 4, 5). Series **1b**, **1d** and **1e** also follow this regularity. It is possible that acyclic α-oxoketene *S,S*-acetal molecules were less stable than cyclic ones. It is demonstrated from Table 3 that the reaction status of α-oxoketene *S,S*-acetals with naphthenic base, methyl and butyl was not as efficient as of α-oxoketene S,S-acetals with phenyl groups. From Table 3, it was found that **1a** and **1c** bear phenyls while the others do not, and the lone pair electrons on N can form p-π conjugation with the phenyls which disperses the electronic density of the N atom. Without enough electron cloud, the N atom was more resistant to nucleophilic attack by the carbonium ion of halohydrocarbon followed by a Hofmann alkylation reaction mechanism [29,30]. The side reactions of **1a** and **1c** were reduced while for **1b**, **1d** and **1e** no phenyl p-π conjugation is possible. Using the optimized experimental procedures the results also revealed that the series **1b**, **1d** and **1e** were too sensitive to variations. The reaction always led to poor results, even if the temperature was slightly higher. Therefore, different structural skeletons have different effects on this reaction. Further investigations are necessary to analyze this in more detail.

**Scheme 1:**
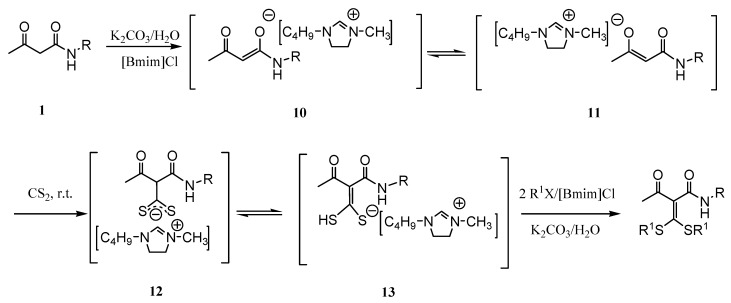
A tentative mechanism of [Bmim]Cl catalytic synthesis of α-oxoketene *S,S-*acetals.

On the basis of these results, in combination with a former study [8], a tentative mechanism of [Bmim]Cl catalytic synthesis of α-oxoketene *S,S*-acetals was shown in Scheme 1. Enolates **10** and **11** can be derived from deprotonation of 1,3-dicarbonyl compounds in the present of [Bmim]Cl/H_2_O by a weak base. Then enolates reaction with CS_2_ resulted in **12** and **12** isomerized to give the unsaturated monothiolate anion **13**. Compound **13** undergoes the *S*-alkylation with the halohydrocarbon to yield the product α-oxoketene *S,S-*acetal.

## 3. Experimental

### 3.1. Preparation of Materials

All reagents used were purchased from the Lanzhou Institute of Chemical Physics, China National Medicines Corporation Ltd and other commercial suppliers and were used as supplied unless otherwise stated. TLC was carried out on a Merck Kieselgel GF254. Purification or separation of product was carried out with flash column chromatography using 300–400 mesh silica gel. ^1^H-NMR and ^13^C-NMR spectra were determined using TMS as an internal reference with a Bruker Avance-300 NMR spectrometer operating at 300 MHz and 75 MHz, respectively. MS analysis was carried out on an API-3000 LC-MS-MS instrument.

*N*-Cyclohexylacetoacetamide/*N*-Butyl-3-oxo-butanamide was synthesized according to the previous literature method [31]. Diketene (10 mmol) was added dropwise in a mixture of cyclohexylamine/1-butylamine (10 mmol) and benzene or water (20 mL) over 1 hour at 0 °C. Then the resulting mixture was stirred for 2 h at room temperature. After the reaction completed as indicated by TLC, extraction of the resulting mixture and purification the crude product furnished *N*-cyclohexylacetoacetamide/*N*-butyl-3-oxo-butanamide in 94%/89% yield.

### 3.2. General Procedure for the Preparation of α-oxoketene S,S-acetals ***6a**-**7a***, ***7b-9b*** and ***6c-7c***

1,3-Dicarbonyl compounds (5 mmol) and [Bmim]Cl (20 mmol) were added to water (15 mL) at 0 °C. After stirred for 3 min, potassium carbonate (12.5 mmol) was added and then the mixture was stirred for an hour at 0 °C. Then carbon disulfide (6 mmol) was added, and the mixture was stirred for another hour and then halohydrocarbon [5 mmol, 1,3-dibromopropane (**6a** and **6c**), 5 mmol 1,2-dibromoethane (**7a**, **7b** and **7c**), 10 mmol bromoethane (**8b**) or 10 mmol iodomethane (**9b**)] was added dropwise over 30 min, then the resulting mixture was stirred for about 5-6 hours at room temperature. Because the product was not dissolved in the [Bmim]Cl/H_2_O system and acted as solid state, the product was filtrated and washed with water and the residual liquid could be reused without any treatment. After drying under vacuum for 5 hours, a crude solid was obtained. Purification by column chromatography afforded the product as a yellowish or white solid in moderate yield. All compounds exhibited spectral data consistent with their structures. ^1^H-NMR, ^13^C-NMR and MS spectral data of compounds were shown as below:

*N-Phenyl-2-(1,3-dithian-2-ylidene)-3-oxobutanamide* (**6a**): white solid, ^1^H-NMR (CDCl_3_) δ: 8.14 (s, 1H, -NH-), 7.65(d, *J* = 7.9 Hz, 2H, H-Ph), 7.37 (t, *J* = 7.9 Hz, 2H, H-Ph), 7.16 (t, *J* = 7.4 Hz, 1H, H-Ph), 2.98 (t, *J* = 7.0 Hz, 4H, -CH_2_-), 2.39 (s, 3H, -CH_3_), 2.32–2.16 (m, 2H, -CH_2_-); ^13^C-NMR (CDCl_3_) δ: 193.88, 164.78, 164.27, 137.86, 132.42, 129.12, 124.71, 119.99, 29.35, 29.17, 28.94, 23.98. MS (ESI): [M+H]^+^ 294.1.

*N-Phenyl-2-(1,3-dithiolan-2-ylidene)-3-oxobutanamide* (**7a**): white solid, ^1^H-NMR (CDCl_3_) δ: 8.19 (s, 1H, -NH-), 7.65 (d, *J* = 7.4 Hz, 2H, H-Ph), 7.38 (t, *J* = 7.9 Hz, 2H, H-Ph), 7.17 (t, *J* = 7.4 Hz, 1H, H-Ph), 3.48–3.41 (m, 2H, -CH_2_-), 3.40–3.32 (m, 2H, -CH_2_-), 2.42 (s, 3H, -CH_3_); ^13^C-NMR (CDCl_3_) δ: 192.91, 170.10, 165.19, 137.68, 129.16, 124.82, 123.77, 120.09, 38.54, 36.62, 28.63. MS (ESI): [M+H]^+^ 280.0.

*N-Cyclohexyl-2-(1,3-dithiolan-2-ylidene)-3-oxobutanamide* (**7b**): white solid, ^1^H-NMR (CDCl_3_) δ: 5.84 (d, *J* = 7.8 Hz, 1H, -NH-), 4.04–3.82 (m, 1H, -CH-), 3.47–3.34 (m, 2H, -CH_2_-), 3.34–3.21 (m, 2H, -CH_2_-), 2.28 (s, 3H, -CH_3_), 2.09–1.93 (m, 2H, -CH_2_-), 1.82–1.67 (m, 2H, -CH_2_-), 1.65–1.09 (m, 6H, -CH_2_-CH_2_-CH_2_-); ^13^C-NMR (CDCl_3_) δ: 191.76, 167.13, 166.58, 124.19, 48.78, 38.68, 36.29, 32.82, 27.86, 25.53, 24.78. MS (ESI): [M+H]^+^ 286.1.

*N-Cyclohexyl-2-(bis(ethylthio)methylene)-3-oxobutanamide* (**8b**): white solid, ^1^H-NMR (CDCl_3_) δ: 6.05 (d, *J* = 7.1 Hz, 1H, -NH-), 3.91–3.77 (m, 1H, -CH-), 2.88 (q, *J* = 7.4 Hz, 4H, -S-CH_2_-), 2.39 (s, 3H, -CO-CH_3_), 2.07–1.85 (m, 2H, -CH_2_-), 1.70–0.98 (m, 8H, -CH_2_-CH_2_-CH_2_-CH_2_-), 1.27 (t, *J* = 7.4 Hz, 6H, -CH_3_); ^13^C-NMR (CDCl_3_) δ: 197.76, 164.04, 147.26, 142.22, 48.61, 32.78, 30.13, 29.29, 25.53, 24.73, 14.76. MS (ESI): [M+H]^+^ 316.1.

*N-Cyclohexyl-2-(bis(methylthio)methylene)-3-oxobutanamide* (**9b**): white solid, ^1^H-NMR (CDCl_3_) δ: 5.99 (d, *J* = 7.2 Hz, 1H, -NH-), 3.93–3.76 (m, 1H, -CH-), 2.41 (s, 6H, -S-CH_3_), 2.37 (s, 3H, -CH_3_), 1.95 (dd, *J* = 12.8, 3.3 Hz, 2H, -CH_2_-), 1.78–1.51 (m, 4H, -CH_2_-CH_2_-), 1.47–1.02 (m, 6H, -CH_2_-CH_2_-CH_2_-); ^13^C-NMR (CDCl_3_) δ: 196.94, 164.33, 152.86, 139.40, 48.67, 32.77, 29.79, 25.53, 24.74, 18.81, 17.81. MS (ESI): [M+H]^+^ 288.1.

*N-(2-Methylphenyl)-2-(1,3-dithian-2-ylidene)-3-oxobutanamide* (**6c**): white solid, ^1^H-NMR (CDCl_3_) δ: 8.00 (d, *J* = 8.3 Hz, 1H, H-Ph), 7.88 (s, 1H, -NH-), 7.26–7.17 (m, 2H, H-Ph), 7.10 (t, *J* = 7.5 Hz, 1H, H-Ph), 3.00 (t, *J* = 6.9 Hz, 4H, -CH_2_-), 2.44 (s, 3H, -CO-CH_3_), 2.32 (s, 3H, CH_3_-Ph), 2.31–2.21 (m, 2H, -CH_2_-); ^13^C-NMR (CDCl_3_) δ: 194.75, 164.54, 163.96, 135.55, 132.28, 130.57, 128.92, 126.84, 125.33, 122.61, 29.41, 28.98, 24.08, 18.00. MS (ESI): [M+H]^+^ 308.1.

*N-(2-Methylphenyl)-2-(1,3-dithiolan-2-ylidene)-3-oxobutanamide* (**7c**): white solid, ^1^H-NMR (CDCl_3_) δ: 8.08 (s, 1H, H-Ph), 7.96 (d, *J* = 6.5 Hz, 1H, -NH-), 7.26–7.20 (m, 2H, H-Ph), 7.15–7.04 (m, 1H, H-Ph), 3.52–3.42 (m, 2H, -CH_2_-), 3.41–3.32 (m, 2H, -CH_2_-), 2.47 (s, 3H, -CO-CH_3_), 2.34 (s, 3H, CH_3_-Ph); ^13^C-NMR (CDCl_3_) δ: 193.33, 170.13, 165.16, 135.55, 130.64, 129.36, 126.84, 125.46, 123.68, 122.87, 38.45, 36.71, 28.87, 18.11. MS (ESI): [M+H]^+^ 294.1.

### 3.3. General Procedure for the Preparation of α-oxoketene S,S-acetals ***7d**-**9d*** and ***7e**-**9e***

1,3-Dicarbonyl compounds (5 mmol) and [Bmim]Cl (20 mmol) were added into water (15 mL) at 0 °C. After stirring for 3 min, potassium carbonate (12.5 mmol) was added and then the mixture was stirred for another hour at 0 °C. Then carbon disulfide (6 mmol) was added, and the mixture was stirred for another hour and then halohydrocarbon [5 mmol, 1,2-dibromoethane (**7d** and **7e**), 10 mmol bromoethane (**8d** and **8e**) or 10 mmol iodomethane (**9d** and **9e**)] was added dropwise over 30 min, then the resulting mixture was stirred for about 6 hours at room temperature. Because the product was dissolved in the [Bmim]Cl/H_2_O system and acted as liquid state, the product was extracted by ether for four times and the residual liquid could be reused without any treatment. Purification with column chromatography afforded the product as a yellowish solid or yellow oil in moderate yield.

*N-Methyl-2-(1,3-dithiolan-2-ylidene)-3-oxobutanamide* (**7d**): yellowish solid, ^1^H-NMR (CDCl_3_) δ: 6.14 (s, 1H, -NH-), 3.47–3.26 (m, 4H, -CH_2_-), 2.97 (d, *J* = 4.9 Hz, 3H, CH_3_-N), 2.32 (s, 3H, -CH_3_); ^13^C-NMR (CDCl_3_) δ: 192.22, 168.17, 123.73, 38.62, 36.35, 28.15, 26.71. MS (ESI): [M+H]^+^ 218.0.

*N-Methyl-2-(bis(ethylthio)methylene)-3-oxobutanamide* (**8d**): yellow solid, ^1^H-NMR (CDCl_3_) δ: 6.31 (s, 1H, -NH-), 2.90 (q, *J* = 7.4 Hz, 4H, -CH_2_-), 2.89 (d, *J* = 4.9 Hz, 3H, CH_3_-N), 2.43 (s, 3H, -CO-CH_3_), 1.29 (t, *J* = 7.4 Hz, 6H, -CH_3_); ^13^C-NMR (CDCl_3_) δ: 198.52, 165.28, 147.45, 141.76, 30.40, 29.21, 26.56, 14.69. MS (ESI): [M+H]^+^ 248.1.

*N-Methyl-2-(bis(methylthio)methylene)-3-oxobutanamide* (**9d**): yellow solid, ^1^H**-**NMR (CDCl_3_) δ: 6.25 (s, 1H, -NH-), 2.88 (d, *J* = 4.9 Hz, 3H, N-CH_3_), 2.40 (s, 3H, -S-CH_3_), 2.40(s, 3H, -S-CH_3_), 2.39 (s, 3H, -CO-CH_3_); ^13^C-NMR (CDCl_3_) δ: 197.78, 165.54, 152.96, 138.93, 30.07, 26.62, 18.17. MS (ESI): [M+H]^+^ 220.0.

*N-(n-Butyl)-2-(1,3-dithiolan-2-ylidene)-3-oxobutanamide* (**7e**): yellowish solid, ^1^H-NMR (CDCl_3_) δ: 6.09 (s, 1H, -NH-), 3.48–3.23 (m, 6H, -CH_2_-), 2.28 (s, 3H, -CO-CH_3_), 1.65–1.52 (m, 2H, -CH_2_-), 1.49–1.29 (m, 2H, -CH_2_-), 0.93 (t, *J* = 7.3 Hz, 3H, -CH_3_); ^13^C-NMR (CDCl_3_) δ: 192.00, 167.61, 167.50, 124.01, 39.80, 38.65, 36.33, 31.44, 28.01, 20.23, 13.75. MS (ESI): [M+H]^+^ 260.1.

*N-(n-Butyl)-2-(bis(ethylthio)methylene)-3-oxobutanamide* (**8e**): yellow oil, ^1^H-NMR (CDCl_3_) δ: 6.24 (s, 1H, -NH-), 2.07 (q, *J* = 5.9 Hz, 2H, N-CH_2_-), 2.95–2.80 (m, 4H, -S-CH_2_-), 2.40 (s, 3H, -CO-CH_3_), 1.53–1.45 (m, 2H, -CH_2_-), 1.38–1.31 (m, 2H, -CH_2_-), 1.30–1.21 (m, 6H, -CH_2_-), 0.91 (t, *J* = 6.9 Hz, 3H, -CH_3_); ^13^C-NMR (CDCl_3_) δ: 198.30, 164.67, 147.07, 142.12, 39.63, 31.37, 30.31, 29.19, 20.15, 14.72, 13.71. MS (ESI): [M+H]^+^ 290.1.

*N-(n-Butyl)-2-(bis(methylthio)methylene)-3-oxobutanamide* (**9e**): yellow oil, ^1^H**-**NMR (CDCl_3_) δ: 6.22 (s, 1H, -NH-), 3.34–3.30 (m, 2H, N-CH_2_-), 2.40–2.36 (m, 9H, -CO-CH_3_, -S-CH_3_), 1.53–1.48 (m, 2H, -CH_2_-), 1.37–1.32 (m, 2H, -CH_2_-), 0.94–0.87 (m, 3H, -CH_3_); ^13^C-NMR (CDCl_3_) δ: 197.44, 165.02, 152.80, 139.23, 39.69, 31.32, 29.95, 20.15, 18.19, 13.72. MS (ESI): [M+H]^+^ 262.1.

## 4. Conclusions

In conclusion, a clean, environmentally friendly method has been developed for the synthesis of α-oxoketene *S,S*-acetals in moderate yields based on the reaction of 1,3-dicarbonyl compounds with carbon disulfide and halohydrocarbon in the presence of potassium carbonate in a [Bmim]Cl/water homogeneous system. A simple procedure, mild conditions, moderate yields, multiple recycling of wet ionic liquid and environmental tolerability make the present protocol highly attractive for academic research and practical applications.

## Figures and Tables

**Table 1:**
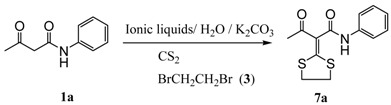
Reaction of **1a** with carbon disulfide and 1,2-dibromoethane in ionic liquids/water under different conditions.

Entry	Reaction Medium	Amount ^a^ (mmol)/(mL)	K_2_CO_3_ (mmol)	1a (mmol)	Time ^b^ (h)	Yield ^c^ (%)
1	H_2_O	15 (mL)	12.5	5	20	0
2	[EPy]BF_4_	50 (mmol)	12.5	5	20	trace
3	[Bmim]Cl	50 (mmol)	12.5	5	20	trace
4	[EPy]BF_4_/H_2_O	50/15	12.5	5	15	71
5	[Bmim]Cl/H_2_O	50/15	12.5	5	5	73
6	[Bmim]Cl/H_2_O	40/15	12.5	5	5	74
7	[Bmim]Cl/H_2_O	30/15	12.5	5	5	73
8	[Bmim]Cl/H_2_O	20/15	12.5	5	5	74
9	[Bmim]Cl/H_2_O	10/15	12.5	5	8	65
10	[Bmim]Cl/H_2_O	5/15	12.5	5	11	61
11	[Bmim]Cl/H_2_O	0.1/15	12.5	5	20	43

^a^ Amount of reaction medium; ^b^ The reaction time was recorded after the addition of 1,2-dibromoethane; ^c^ Yields of **7a** were obtained by purification with column chromatography.

**Table 2:** Recycling of [Bmim]Cl/water in synthesis of **7a**.

Entry	Reuse times	Reaction medium	1a (mmol)	CS_2_ (mmol)	BrCH_2_CH_2_Br (mmol)	Time ^a^ (h)	Yield ^b^ (%)
1	2	[Bmim]Cl/H_2_O ^c^	5	6	5	5	70
2	3	[Bmim]Cl/H_2_O ^d^	5	6	5	6	65
3	4	[Bmim]Cl/H_2_O ^e^	5	6	5	6	65
4	5	[Bmim]Cl/H_2_O ^f^	5	6	5	7	66

^a^ The reaction time was recorded after the addition of 1,2-dibromoethane; ^b^ Yields of 7a were obtained by purification with column chromatography; ^c^ [Bmim]Cl/H_2_O was obtained after filtration the product of Table 1 Entry 8 and without any treatment; ^d^ [Bmim]Cl/H_2_O was obtained after filtration the product of Table 2 Entry 1 and without any treatment; ^e^ [Bmim]Cl/H_2_O was obtained after filtration the product of Table 2 Entry 2 and without any treatment; ^f^ [Bmim]Cl/H_2_O was obtained after filtration the product of Table 2 Entry 3 and without any treatment.

**Table 3:**
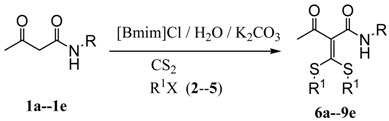
Preparation of α-oxoketene S,S-acetals **6a-9e** from 1,3-dicarbonyl compounds **1** in [Bmim]Cl/H_2_O.

Entry	Substrate			Substrate			Time ^a^ (h)	Product	Yield ^b^ (%)
	1 R		2–5	X R^1^		R^1^			
1	**1a**	C_6_H_5_	**2**	Br	-(CH_2_)_3_-		5	**6a**	73
2	**1a**	C_6_H_5_	**3**	Br	-(CH_2_)_2_-		5	**7a**	74
3	**1b**	CH(CH_2_)_5_	**3**	Br	-(CH_2_)_2_-		6	**7b**	60
4	**1b**	CH(CH_2_)_5_	**4**	Br	-C_2_H_5_	-C_2_H_5_	6	**8b**	55
5	**1b**	CH(CH_2_)_5_	**5**	I	-CH_3_	-CH_3_	6	**9b**	53
6	**1c**	o-Methylphenyl	**2**	Br	-(CH_2_)_3_-		6	**6c**	70
7	**1c**	o-Methylphenyl		**3**	Br	-(CH_2_)_2_-	6	**7c**	72
8	**1d**	-CH_3_	**3**	Br	-(CH_2_)_2_-		6	**7d**	65
9	**1d**	-CH_3_	**4**	Br	-C_2_H_5_	-C_2_H_5_	6	**8d**	61
10	**1d**	-CH_3_	**5**	I	-CH_3_	-CH_3_	6	**9d**	60
11	**1e**	-(CH_2_)_3_CH_3_	**3**	Br	-(CH_2_)_2_-		6	**7e**	66
12	**1e**	-(CH_2_)_3_CH_3_	**4**	Br	-C_2_H_5_	-C_2_H_5_	6	**8e**	55
13	**1e**	-(CH_2_)_3_CH_3_	**5**	I	-CH_3_	-CH_3_	6	**9e**	58

^a^ The reaction time was recorded after the addition of halohydrocarbon; ^b^ Yields of **6a-9e** obtained by purification with column chromatography.
